# Prenatal prediction and typing of placental invasion using MRI deep and radiomic features

**DOI:** 10.1186/s12938-021-00893-5

**Published:** 2021-06-05

**Authors:** Rongrong Xuan, Tao Li, Yutao Wang, Jian Xu, Wei Jin

**Affiliations:** 1 Affiliated Hospital of Medical School, Ningbo University, Ningbo, 315020 Zhejiang China; 2 Faculty of Electrical Engineering and Computer Science, Ningbo University, Ningbo, 315211 Zhejiang China; 3 Ningbo Women’s and Children’s Hospital, Ningbo, 315012 Zhejiang China

**Keywords:** Placental invasion, Radiomics, Deep learning, MRI, Assistant diagnosis

## Abstract

**Background:**

To predict placental invasion (PI) and determine the subtype according to the degree of implantation, and to help physicians develop appropriate therapeutic measures, a prenatal prediction and typing of placental invasion method using MRI deep and radiomic features were proposed.

**Methods:**

The placental tissue of abdominal magnetic resonance (MR) image was segmented to form the regions of interest (ROI) using U-net. The radiomic features were subsequently extracted from ROI. Simultaneously, a deep dynamic convolution neural network (DDCNN) with codec structure was established, which was trained by an autoencoder model to extract the deep features from ROI. Finally, combining the radiomic features and deep features, a classifier based on the multi-layer perceptron model was designed. The classifier was trained to predict prenatal placental invasion as well as determine the invasion subtype.

**Results:**

The experimental results show that the average accuracy, sensitivity, and specificity of the proposed method are 0.877, 0.857, and 0.954 respectively, and the area under the ROC curve (AUC) is 0.904, which outperforms the traditional radiomic based auxiliary diagnostic methods.

**Conclusions:**

This work not only labeled the placental tissue of MR image in pregnant women automatically but also realized the objective evaluation of placental invasion, thus providing a new approach for the prenatal diagnosis of placental invasion.

## Background

Placental invasion (PI) is a phenomenon that placental villi directly invade the myometrium due to abnormal hyperplasia of decidua [1]. According to the degree of implantation, it can be divided into three types: placenta accrete (PA), placenta increta (PC), and placenta percreta (PP) [2]. It is called PA if the placental villi are directly attached to the myometrium and require manual placental dissection during delivery, and when the placental villi penetrate deep into the uterine myometrium, it is called PC. In the most severe case, if the placental villi can reach the serous layer, or even penetrate the serosa layer, to the bladder or rectum, it is called PP. It may cause different degrees of damage to pregnant women according to the severity of placenta implantation. The harm of the occurrence of PI is mild to difficult to peel the placenta during delivery, severe to postpartum hemorrhage, amniotic fluid embolism, diffuse intravascular coagulation (DIC), and even seriously endanger the life of pregnant women. There are many high-risk factors for PI, including the history of cesarean section, placenta previa, multiple abortions and curettage, hysteromyomectomy, and other uterine-related operations, and the advanced age of pregnant women, etc [3, 4]. Among these factors, the history of cesarean section is the main risk factor for placental implantation.

In recent years, with the increase of cesarean section and abortion operations, the incidence of PI has been increasing year by year [5]. Due to the clinical symptoms of placental invasion are not obvious and lack of specificity before delivery, it is very difficult to diagnose by clinical manifestations. At present, the color Doppler ultrasound (US) and magnetic resonance imaging (MRI) are commonly used in clinical diagnosis and classification of prenatal placenta implantation. Among them, ultrasound examination has many advantages, such as low price, wide application, harmless to the mother and child, etc. It is the preferred imaging method for the diagnosis of placental implantation. However, the detection rate of PI by ultrasound will be reduced or even difficult to detect when the placenta is located at the fundus or posterior wall of the uterus, or there are interfering factors such as intestinal gas. Moreover, the ultrasound examination is of limited value in assessing the degree of placental invasion [6]. MRI examination of the placenta is not interfered by maternal body size, intestinal gas, or placental position, and has a large field of view and high soft tissue resolution. It can be an important complementary imaging method when ultrasound diagnosis of placental implantation is uncertain or limited in evaluation, especially when evaluating the degree of placental implantation and its infiltration to the organs around the uterus [6, 7]. At present, the diagnosis of placental invasion with MRI mainly depends on the visual interpretation of clinicians. This method not only relies on the experience of clinicians, but also easily interfered by various subjective and objective factors, and its efficiency is not high. For the computer-aided diagnosis of PI methods based on radiomics, professional radiologists should label ROI manually, then high-throughput features should be extracted based on ROI, and identified PI by traditional machine learning finally. Sun et al. [8] analyzed 9 pregnant women with pathologically confirmed placental invasion and 56 patients with simple placenta previa using the radiomics approach. They initially extracted texture features from the patient’s original MRI images and Laplace Gaussian (LOG) filtered MRI images. Then, the proposed texture features are predicted using an automatic machine learning algorithm. Finally, the intra-placental texture features were shown to be highly efficient in predicting placental invasion after 24 weeks of gestation. Similarly, Romeo et al. [9] explored whether MRI texture features could help to assess the presence of PI in patients with placenta previa. They first manually located ROI on sagittal or coronal T2 weighted images. Then, texture features in the ROI region are extracted by radiomics. Finally, the machine learning model is established to train and test with the extracted features. Among all machine learning algorithms, the *k*-nearest neighbor algorithm had the highest accuracy of 98.1%. The experimental results suggest that machine learning analysis using MRI-derived texture features is a feasible tool for identifying placental tissue abnormalities in patients with placenta previa. Although the method of radiomics is widely used, it also has some shortcomings. Such methods are difficult to obtain high-quality annotation data and the extracted features lack the ability to express higher-order semantic information, resulting in high missed diagnosis and misdiagnosis when faced with complex cases [10].

Recently, deep learning methods have been widely used in medical image analysis, such as segmentation and disease computer-aided diagnosis [11–17]. In particular, the related technology represented by U-net has achieved excellent performance in medical segmentation [18]. The U-net consists of a contracting path and an expansive path. In the contracting path, the image features of different levels are extracted by repeated convolution and pooling. And in the expansive path, through the up-sampling and convolution operation symmetrical to the contracting path, and combining the shallow positioning information and the deep classification information of the objects using jump connection, the U-net can fully acquire multi-level features of the image and simultaneously achieves accurate object positioning. Experiments show that U-net based methods have been successful in many medical image segmentation tasks [19–22].

On the other hand, due to the advantages of the local receptive field, weight sharing, and temporal & spatial sub-sampling, the deep convolution neural network (CNN) can realize the invariance of displacement, scale, and deformation to some extent, and can mine the semantic information contained in the images. Therefore, based on the deep features extracted by CNN, deep learning has been widely used in medical image aided diagnosis. At present, in medical image processing, deep learning and radiomics are also showing a trend of mutual integration and collaborative development. A common kind of method is the feature-level fusion, which combines the deep features and radiomics features into a new feature vector for the subsequent disease classification and prediction [15, 23]. This scheme has been applied to tasks such as detection and classification of lung nodules [24, 25], image attribute analysis of tumors [26], and prediction of cancer survival rates [27]. In July 2019, Zhu et al. [28] used the MRI of 181 patients with meningioma to establish a deep radiomics model to classify meningioma in a non-invasive manner. The results show that the deep learning combined radiomics model has outstanding quantification ability in the non-invasive individualized meningioma grade prediction. Although the model of combining deep learning and radiomics is playing an increasingly important role in medical imaging diagnosis, there is still a lack of relevant research in the auxiliary diagnosis of placental invasion.

Based on the above analysis, deep learning and radiomics were combined to carry out the prenatal diagnosis of placental invasion based on MRI in this paper. Firstly, we train the U-net to segment the placental region of the magnetic resonance (MR) image and extract the radiomics features. Then, we construct a deep dynamic convolution neural network via self-encoder learning to extract the deep features of the placenta tissues to characterize the status of placental invasion. Finally, a multi-layer perceptron network is constructed and trained to realize the prenatal diagnosis of placental invasion and determine the subtypes of placental invasion.

The main contributions of this work are as follows:

1. A new method for the detection of placental invasion based on MRI is proposed.
2. The placental tissue of pregnant women was marked automatically by deep segmentation instead of manual, which provides the regions of interest (ROI) for subsequent diagnosis of placental invasion.
3. The influence of different degrees of placental boundary dilation on the prediction accuracy of placental invasion was quantitatively analyzed.
4. A deep dynamic convolution neural network (DDCNN) with codec structure was established to better characterize the semantic information of different placental implant types.
5. The prenatal diagnosis and accurate typing of placental invasion were realized by fusing the deep and radiomic features.

This paper is organized as follows. In this section, we introduce the definition and related work of placental implantation and briefly describe the characteristics of the methods in this paper. In the next four sections, we first present the experimental configuration and evaluation metrics in the “Results” section and analyze the experimental results. It is followed by a “Conclusion” section where we conclude and discuss future work. After that, we discuss the methods in this paper and summarize their advantages and disadvantages in the “Discussion” chapter. Finally, we provide a detailed description of the dataset and methods in the “Methods” section.

## Results

All the experiments were conducted on an AMD Ryzen 7 3800X @ 3.89 GHz with 32-GB RAM. Unless otherwise specified, for all deep learning models, we initialized the weights with random values, set the batch size to 8, set the learning rate to 0.001, and trained 200 epochs on 11G NVIDIA RTX 2080Ti GPU, SGD as the optimizer.

The automatic segmentation of placental tissues in MR images is realized by the trained U-net. The performance of the segmentation method was evaluated quantitatively using the four widely used evaluation metrics, i.e, segmentation Accuracy (ACC), Precision (PRE), Recall (REC), and F1 score (F1). These evaluation metrics were calculated as follows:

1 $$\begin{aligned} {\text{ACC}}&= \frac{{\text{TP}}+{\text{TN}}}{{\text{TP}}+{\text{TN}}+{\text{FP}}+{\text{FN}}} \end{aligned}$$

2 $$\begin{aligned} {\text{PRE}}&= \frac{{\text{TP}}}{{\text{TP}}+{\text{FP}}} \end{aligned}$$

3 $$\begin{aligned} {\text{REC}}&= \frac{{\text{TP}}}{{\text{TP}}+{\text{FN}}} \end{aligned}$$

4 $$\begin{aligned} F_1&= \frac{2*{\text{PRE}}*{\text{REC}}}{{\text{PRE}}+{\text{REC}}} \end{aligned}$$

where *TP*(True Positive) was the number of placenta pixels that were correctly identified as placenta and *FP*(False Positive) was the number of background pixels that were incorrectly identified as placenta. *FN*(False Negative) was the number of placenta pixels that were incorrectly identified as background and *TN*(True Negative) was the number of background pixels that were correctly identified as background.

To evaluate the performance of various methods for predicting and typing placental invasion, the three evaluation metrics, i.e, Average Accuracy (AACC), Average Sensitivity (ASEN), and Average specificity (ASPE) were calculated as follows:

5 $$\begin{aligned} {\text{AACC}}&= \frac{1}{4} * \sum _{i=0}^3{\frac{{\text{TP}}_i+{\text{TN}}_i}{{\text{TP}}_i+{\text{TN}}_i+{\text{FP}}_i+{\text{FN}}_i }} \end{aligned}$$

6 $$\begin{aligned} {\text{ASEN}}&= \frac{1}{4} * \sum _{i=0}^3{\frac{{\text{TP}}_i}{{\text{TP}}_i+{\text{FN}}_i}} \end{aligned}$$

7 $$\begin{aligned} {\text{ASPE}}&= \frac{1}{4} * \sum _{i=0}^3{\frac{{\text{TN}}_i}{{\text{FP}}_i+{\text{TN}}_i}} \end{aligned}$$

where i = 0,$\ldots$, 3 indicate the type of no placental invasion, placenta accreta, placenta increta, and placenta percreta respectively. ${\text{TP}}_i$ is the number of patients that belong to the ith type of placental invasion and were classified exactly as ith type by the classifier. ${\text{FP}}_i$ is the number of patients that do not belong to the ith type of placental invasion but were classified as ith type by the classifier. The definitions of ${\text{TN}}_i$ and ${\text{FN}}_i$ follow this pattern.

### Automatic segmentation of placenta

**Fig. 1:**
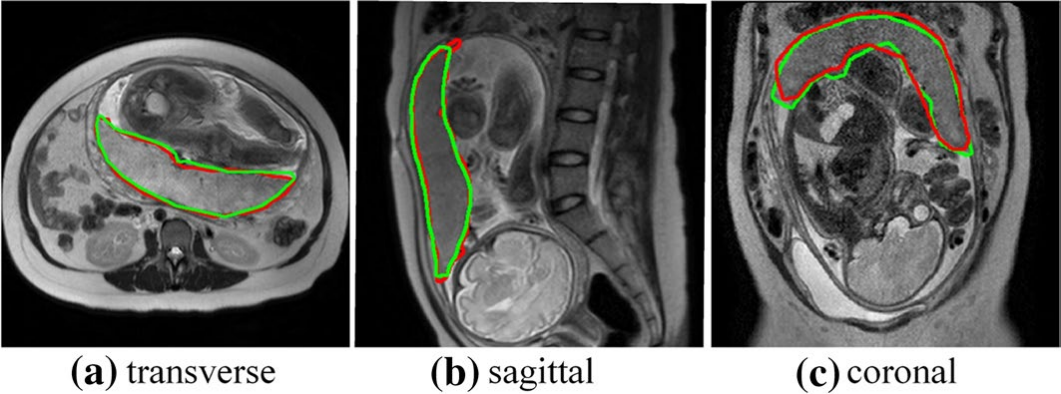
Comparison between u-net automatic segmentation and expert segmentation

We selected 490 T2-sequence MR images with placenta marked by the radiologists as training samples, including 165 transverse, 182 sagittal, and 143 coronal images to train U-net. And the trained U-net was used to segment the placenta automatically on abdominal T2WI MR images. The segmentation results of placental tissue of some test images were shown in Fig. 1. Where Fig. 1a–c were the segmentation results of the transverse, sagittal, and coronal planes respectively.

In the figure, the red border is the boundary of placental tissue delineated by radiologists, and the green border is the boundary of placental tissue automatically segmented by the trained U-net. It can be seen that U-net segmentation results of the transverse and sagittal planes are consistent with the radiologists’ segmentation results. While for the coronal plane, the inconsistency of the segmentation results between the U-net and radiologists are more obvious than that of the transverse and sagittal planes. In general, the segmentation errors of U-Net are all within the controllable range and have little impact on subsequent tasks.

**Fig. 2:**
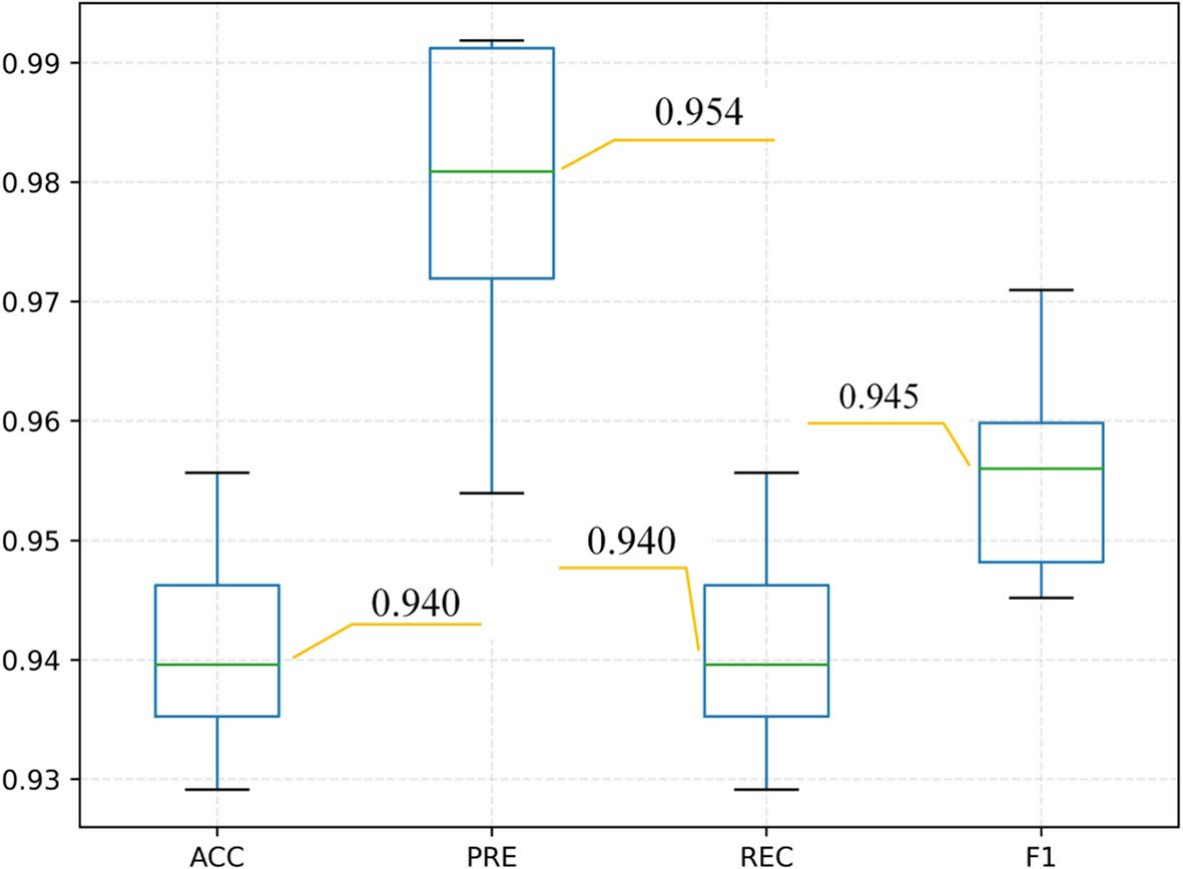
Box plot of accuracy, precision, recall, and F1 score of the placenta segmentation model

To evaluate the performance of the ROI extraction network, we calculated the quantitative indicators of the segmentation results of the test images and gave a statistical box plot as shown in Fig. 2.

The digits in Fig. 2 represent the median of the corresponding metrics. It can be seen that the accuracy and recall of segmentation are both 0.940, the precision is 0.954, and the F1 score is 0.945. Besides, we calculated the inference speed of U-net, whose average computation time of single-image is 29.640ms. The above results show that the model has high segmentation accuracy and can replace radiologists to segment the placental tissue region of the MR image to a certain extent.

### The influence of different pixels extended from the placental region on the accuracy of predicting placental invasion

Normally, the placenta and uterine myometrium are separated by the basal decidua. If the basal decidua is lost due to various reasons, the placental villi will adhere directly to the uterine myometrium in the absence of basal decidua which will lead to placental invasion, and the depth of the invasion of placental villi into the myometrium will determine the severity of placental implantation. Therefore, we extend different pixels from the placental region to form ROIs for subsequent placental invasion diagnosis. To optimize the extension size, we extended the placental region of the T2WI MRI image to the surrounding area with 10, 20, 40, 60 pixels to form ROIs and extracted the radiomic features and deep features from the ROIs. The classification model described in prenatal prediction and typing of placental invasion was used to predict the type of placental invasion, and the performance of different size boundary expansion was evaluated from the aspects of AACC, ASEN, and ASPE. The experimental results are shown in Table 1.

**Table 1:** Performance analysis of the model in different extended pixels

Extended Pixels	AACC	ASEN	ASPE
0	0.834	0.786	0.937
10	0.847	0.798	0.942
20	0.859	0.840	0.947
40	0.877	0.857	0.954
60	0.865	0.848	0.949

It can be seen from the table that the model trained by the samples with the 40 pixels extension from the placental region performed better in various quantitative indicators than other extension models, the AACC, ASEN, and ASPE are 0.877, 0.857, and 0.954 respectively. The results show that the extension of the placental tissue region to form ROIs is favorable to detect placental invasion, and the border extension with 40 pixels is relatively good. The following experiments for the computer-assisted diagnosis of placental invasion are all based on the ROIs formed after the 40 pixels extension of the placental region.

### The diagnostic ability of the proposed approach

To objectively evaluate the clinical application potential of the proposed method in the assisted diagnosis of placental invasion, we tested the model with the test set. The confusion matrix of the diagnosis results for the test set is shown below.

**Table 2:** Confusion matrix of the diagnosis results of the proposed method for typing of placental invasion

True label	Typing as			
	No invasion	Accreta	Increta	Percreta
No invasion	62	0	1	0
Accreta	4	21	7	0
Increta	3	4	50	0
Percreta	0	0	1	10

It can be seen from Table 2 that the model has a strong ability to distinguish between non-placental invasion (normal placenta) and placenta percreta, which is consistent with the signs of placental invasion in MR images. The image features of non-placental invasion and placental percreta are more obvious than those of placental accreta and increta. Due to the clinical manifestations of the difference between placenta accreta and placenta increta are ambiguous, the model has certain room for improvement in the ability to distinguish placental increta and placenta accreta. On the other hand, a large number of studies have also confirmed there is little difference between placental accreta and increta, and it is difficult to completely distinguish them by imaging alone. For this difficulty, other clinical information of patients can be considered in the next step to further improve the discriminatory ability of the model.

### Comparisons with other approaches

Various approaches can be used for the diagnosis of placental invasion. Each approach has its respective accuracy level. Here, the proposed approach was compared with traditional methods based on machine learning (ML) and deep learning (DL). The comparison of traditional machine learning methods includes random forest (RF), decision tree (DT), and logistic regression (LR), which use only radiomics features for placental invasion identification and typing. For the random forest, univariate feature selection was used to screen the radiomics features, and the 20 highest-scoring features were selected to train the random forest. All machine learning models are built on Scikit-learn (version 0.23, download link: https://scikit-learn.org/stable/index.html). The approaches based on deep learning combine deep features with radiomic features to type placental invasion. Specifically, the proposed approach uses the dynamic convolutional neural network to extract deep features, while the comparison method uses a standard convolutional neural network (CNN) to extract deep features. We also compare with current deep learning methods, including ResNet50 (RN) [29] and SENet (SEN) [30].

The performance of different approaches was evaluated in terms of AUC (area under ROC curve), AACC, ASEN, and ASPE. The results are shown in Table 3.

**Table 3:** Comparison of different approaches for typing placental invasion

Methods		AUC	AACC	ASEN	ASPE	Time/ms
ML						
LR		0.768	0.706	0.677	0.901	0.003
DT		0.780	0.742	0.693	0.911	0.004
RF		0.796	0.767	0.709	0.919	0.037
DL						
RN		0.879	0.847	0.816	0.943	17.845
SEN		0.874	0.834	0.801	0.938	8.472
CNN		0.870	0.840	0.805	0.937	13.306
Ours		0.904	0.877	0.857	0.954	16.140

As can be seen from the table, compared with other traditional machine learning approaches, the random forest has better performance, with an average accuracy of 0.767, an average sensitivity of 0.709, and an average specificity of 0.919. The average accuracy, sensitivity, and specificity of the traditional deep learning approach were 0.840, 0.805, and 0.937, respectively, which improved the performance of traditional machine learning approaches. The average accuracy, sensitivity, and specificity of the proposed approach reached 0.877, 0.857, and 0.954, respectively, and all the evaluation metrics were the best. From the perspective of AUC, the proposed approach is 0.904, which is superior to all other approaches, followed by ResNet50 and SENet with 0.879 and 0.874, respectively. The AUC of the standard convolution neural network is 0.870, while the approaches based on traditional machine learning models are relatively poor. This indicates that it is beneficial to introduce deep features into the computer-assisted diagnosis of placental invasion and that the dynamic convolutional neural network proposed in this paper can more effectively mine the health semantic information contained in MR images, so as to improve the ability to distinguish pathological subtypes of placental invasion.

To evaluate the computational efficiency of different methods, the total reasoning time is calculated for 163 MRI images in the test set. Then the mean time for placental invasion staging on a single MRI image was calculated for each method. As shown in the last column of Table 3, the average computation time of various methods is listed. It can be seen that overall the inference time of the machine learning methods is faster compared to the deep learning methods. For machine learning methods, the fastest is LR with a single sample inference time of 0.003ms, and the slowest is RF with 0.037ms. For the deep learning methods, ResNet50 took the longest time to detect a single MRI image, 17.845ms, followed by this paper’s method and CNN, 16.140 and 13.306ms, respectively. Note that, except for ResNet and SENet, the other methods mentioned above rely on the results of U-Net segmentation, so the inference time calculation of U-Net should also be taken into account during practical use. Besides, machine learning methods do not need to use GPU for computing, while deep learning methods must use GPU for acceleration to obtain faster speedups. Overall, the method proposed in this paper has higher performance and acceptable efficiency for placental invasion detection compared to competing methods.

## Discussion

Placental invasion is a common emergency in obstetrics, which is mainly caused by patients with traumatic endometrial defects and primary decidual hypoplasia. The patients may have a severe postpartum hemorrhage, postpartum placenta retention, uterine perforation, and secondary infection may occur, which seriously endanger the life of pregnant women and fetuses. According to the severity of placental villi invading the myometrium, placenta accreta can be divided into placenta accreta, placenta increta, and placenta percreta. Accurate prediction of the degree of placental invasion helps to provide more effective treatment for patients.

At present, MRI has been widely used in the diagnosis and subtyping of placental invasion (PI), and the effectiveness of MRI in the diagnosis of PI has been verified by an enormous amount of research [7, 31]. Although some researchers have carried out a computer-aided diagnosis of placental invasion, most of the existing work requires radiologists to manually segment the placental region in advance, which not only relies on expert experience and is inefficient. In this paper, a U-net model that can automatically achieve the segmentation of placental tissue is trained, which provides a scalable medical image segmentation method with high segmentation accuracy, and can provide a reliable ROI for the detection and typing of placental invasion.

To the best of our knowledge, most of the current studies on placental invasion are limited to the use of radiomic features [8, 9, 32–34]. Although the radiomic features have good interpretation, their low-level characteristics make it difficult to mine deep health semantic information of images. In this paper, deep learning is introduced to extract the deep features of ROI and combined with radiomic features to improve the accuracy of placental invasion prediction. On the other hand, in general, the deeper the degree of placental invasion, the more serious of placental villi invasion into the myometrium, that is to say, the prediction of placenta accreta is more dependent on the characteristics of the placental boundary area. In this paper, we extend different pixels from the placental region to form ROIs, and the optimal extension size is determined by experiments. The results show that the proposed method greatly improves the prediction performance.

Although the model in this paper has some advantages in the staging of placental invasion, it still has shortcomings. Firstly, since the model in this paper integrates multiple depth models, it requires more hardware resources and training time compared to machine learning methods. Besides, due to the segmentation model U-net used in this paper, its performance relies on a large number of segmentation labels, so it still requires radiologists to annotate the ROI regions when training the segmentation model. In summary, future work will focus on optimizing the model efficiency, getting a better trade-off between efficiency and accuracy, and improving the generalization ability of the model to extend the method of this paper to other medical image processing tasks.

## Conclusion

With the increasing of cesarean section and other intrauterine operations, the incidence of placental invasion has become a common and frequently occurring disease in obstetrics. Different types of placental invasion cause different degrees of injury to pregnant women, and the placental invasion often has no clinical symptoms or symptoms lack of specificity before delivery, so it is difficult to diagnose through clinical manifestations. At present, the placental invasion diagnosis usually requires professional radiologists to mark the ROI first, which is hard and tedious work, and the segmentation standard is difficult to unify. To solve these problems, this paper adopts deep learning to automatically segment placental region to form ROI based on MR image, then placental invasion was diagnosed and typed according to the degree of implantation by combining radiomic and deep features of ROI. The results show that the proposed approach has the potential to predict different degrees of placental invasion and can be used as an auxiliary tool for the clinical diagnosis of placental invasion.

## Methods

**Fig. 3:**
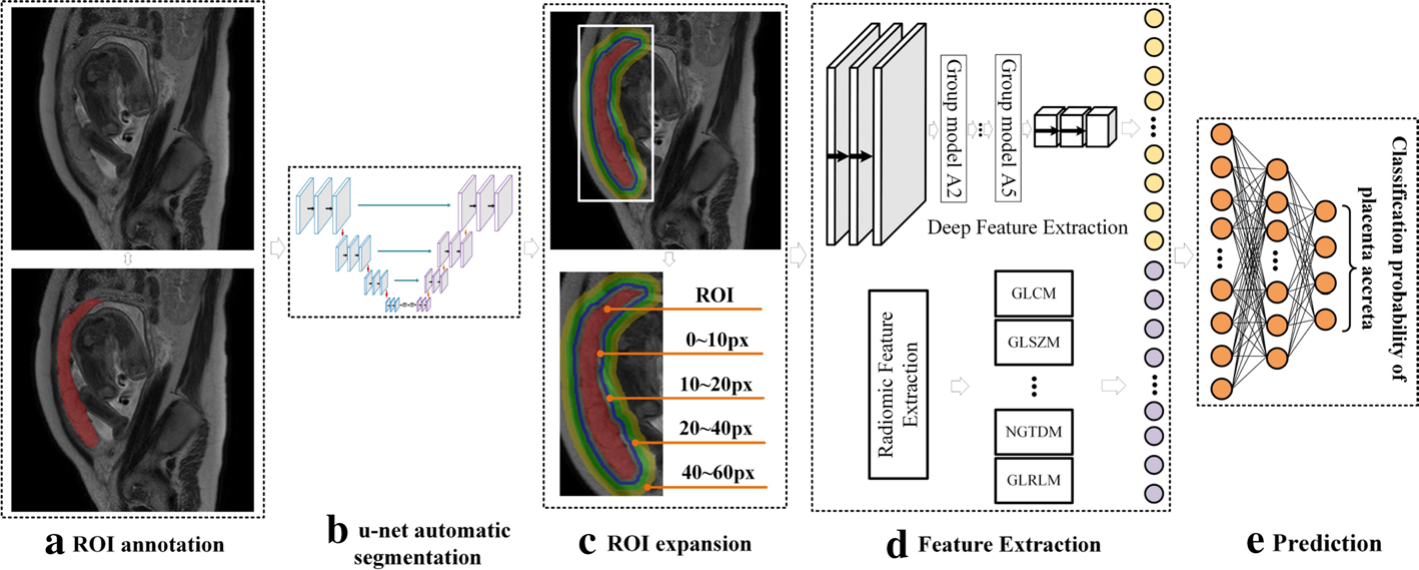
The process flow diagram of this study

To fuse the deep and radiomic features of MRI images, and establish an automatic prenatal prediction and typing model for placental invasion, this research mainly includes data collection, ROI extraction, deep and radiomics features extraction, and classification network training. The process flow diagram is shown in Fig. 3.

In the figure, firstly, the U-net is trained using the ROI data marked by the radiologists to make it have the ability to segment the placental tissue from the original MRI image. Then, the trained U-net is used to realize the automatic extraction of placental tissue, and ROI expansion is performed to determine the relative better ROIso as to the deep features and radiomics features can be extracted. Finally, a multilayer perceptron network is established by combining radiomics and deep features to realize the prenatal diagnosis of placental invasion.

### Data collection

It is fundamental to collect necessary MR images and related clinical materials for the prenatal evaluation of placental invasion. The MR images and clinical materials were collected from the Affiliated Hospital of Medical College of Ningbo University and Ningbo women’s and children’s hospital, the time span of the collected materials was from January 2017 to November 2020. All included cases were suspected of placental invasion by ultrasonography or clinical examination. Meanwhile, the surgical (delivery) and postoperative pathological data of relevant patients were obtained.

All MRI examinations were performed by radiologists with more than 5-year of work experience using 1.5 Tesla units to perform 8 or 16-channel array sensitivity-coded abdominal coil scans. The imaging equipment of the Affiliated Hospital of Medical College of Ningbo University is Ge signa twinspeed 1.5T superconducting dual gradient magnetic resonance scanner with 8-channel body phased array coil. The imaging equipment of Ningbo Women’s and Children’s Hospital is Philips Achieva Noval Dual 1.5T superconducting dual gradient magnetic resonance scanner, using a 16-channel body phased array coil. Before MR examination, the patients were asked to fill the bladder with moderate water and respiratory training. When scanning, the supine position is adopted, and the head is advanced. All sequences are scanned in three directions: transverse, sagittal, and coronal. The scanned images are stored in the hospital’s Picture Archiving and Communication System (PACS) in the Digital Imaging and Communications in Medicine (DICOM) format.

The inclusion criteria were as follows:

(1) Patients who underwent MRI examination after 30 weeks of gestation with T2 weighted image (T2WI) sequence; (2) Patients with definite placental invasion or pathological records after cesarean section; (3) Patients with good image quality and meeting the diagnostic requirements. The exclusion criteria were as follows: (1) Patients without T2WI MRI data; (2) Patients without clinical or surgical pathology confirmation; (3) Patients with severe image artifacts due to fetal movement or poor cooperation of pregnant women.

According to the above criteria, we collected 352 patients’ data from the Affiliated Hospital of Medical College of Ningbo University and Ningbo women’s and children’s Hospital. There were 147 cases without placental invasion and 205 cases with placental invasion. Among 205 cases of placental invasion, 66 cases were placenta accreta, 117 cases were placenta increta and 22 cases were placenta percreta. We divide it into the train set and test set. There were 189 cases in the train set and 163 cases in the test set. In the train set, 84 cases without placental invasion, 34 cases with placenta accrete, 60 cases with placenta increta, and 11 cases with placenta percreta. The test set included 63 cases without placental invasion, 32 cases with placenta accrete, 57 cases with placenta increta, and 11 cases with placenta percreta.

Considering that the main signs of placental invasion in MR imaging are as follows: the placental signal was uneven (low or slightly high, mixed high signal shadows were seen in the placenta) on T2WI images, the local irregular thinning or disappearance of moderate or slightly high signal myometrium on T2WI images, the placenta or (and) uterine localized abnormal protrusion reflected by T2WI images, and low signal strip shadow in the placenta in T2WI images, etc. [32]. Therefore, T2WI is the main reference imaging sequence for clinical diagnosis of placenta accrete. In this study, T2 sequences of transverse, coronal, and sagittal were selected to study the auxiliary diagnosis of placental invasion.

### ROI extraction

**Fig. 4:**
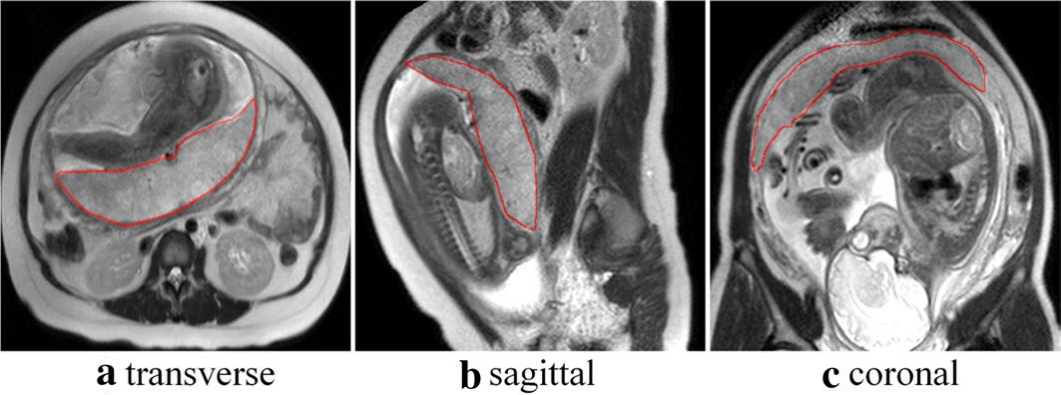
Examples of the T2WI MRI images and labels used in the present study

Extraction of ROI is the basis of computer-aided diagnosis of placental invasion. Based on U-net, we established the model of placental tissue segmentation, thus extracting the ROI automatically. Firstly, some MR images were selected, and two radiologists with more than 5-year working experience annotated the region and of placental tissue and outlined the boundary of placental. The annotation software was ITK-SNAP (version 3.6.0, download website: http://www.itksnap.org/). To ensure annotating placental region accuracy, the two radiologists annotated each image separately and took the intersection of the labeled areas. If the annotating regions of the two radiologists diverge significantly, another radiologist with more than 10-year of working experience was invited to evaluate the labeling results, and the final results were given after negotiation among them. Figure 4 shows the placental tissue boundary of T2WI labeled by the radiologist. Figures (a), (b), and (c) show the transverse, sagittal, and coronal planes respectively.

In this study, 490 T2WI images were selected annotated the placental region as the ground truth training U-net. Recently, U-net has been successfully applied in image segmentation, especially in medical image segmentation. By end-to-end training from very few images, U-net can obtain accurate target boundary location in image segmentation. The U-net consists of two paths: down-sampling and up-sampling. The down-sampling encodes image semantic information through the level by level convolution and pooling while the up-sampling decodes the spatial and multi-scale information by step-by-step de-convolution to acquire multi-level features of the image and simultaneously achieves target segmentation. To make up for the loss of the spatial and boundary information in the encoding stage, the feature maps of the encoder and decoder were fused by concatenating correspondingly using the skip connection. By fusion low-level spatial information and high-level semantic information, the decoder of the U-net can obtain more high-resolution information when up-sampling to recover the details of the original image more perfectly, and then improve the segmentation accuracy.

**Fig. 5:**
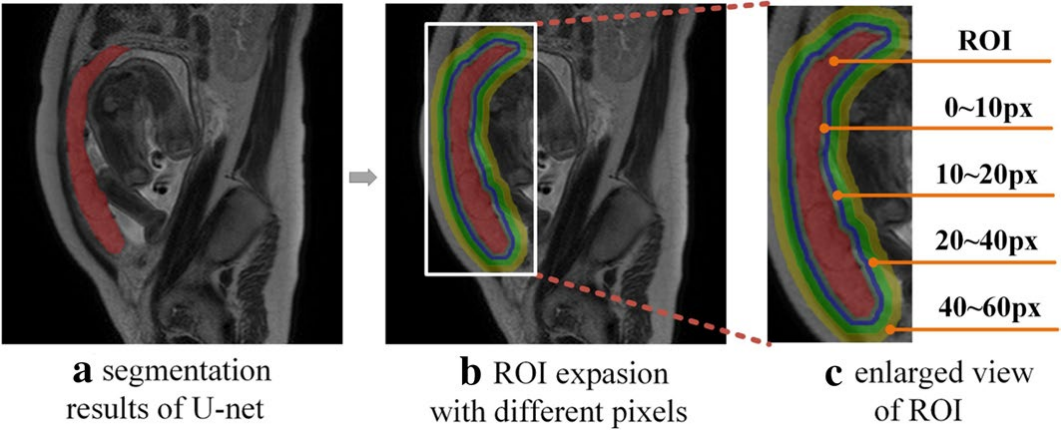
The ROIs formed with the different radial extension on T2WI MRI sagittal plane. The placental tissue is denoted as red, the colored circles denote different radial extensions of the placental tissue

Although U-net can segment the placental area accurately, for subsequent placental invasion typing, placental tissue alone cannot fully characterize the relationship between placenta and neighboring tissues and organs. This is because placental invasion is not only related to the characteristics within the placental region, but also the characteristics of the boundary between the placenta and the uterine myometrium [33]. In addition, according to relevant reports, placental tissue infiltration of the bladder and other tissues and organs adjacent to the placenta is also a specific sign for the diagnosis of penetrating placental invasion [35]. To evaluate the discriminative power of peri-placenta pixels on placental invasion property, 10, 20, 40, and 60 pixels were extended from the placental region segmented by U-Net to form ROIs for subsequent placental invasion diagnosis. The reasonable extension was determined through the follow-up placental invasion diagnosis experiments. Based on ROI, the deep and radiomic features were extracted to construct the evaluation model of placental invasion typing. Figure 5 shows an example of the placental tissue segmented by U-Net and the ROIs formed by extending the boundary with different sizes.

### Extraction of radiomics features

After segmenting the ROI, we use PyRadiomics (version 3.0, download address: https://github.com/Radiomics/pyradiomics) [36] to extract radiomic features to train the auxiliary diagnosis model of placental invasion. The extracted features can be divided into three categories: (1) Intensity-based features; (2) Shape-based features; (3) Texture-based features. Among them, the intensity-based feature transforms ROI into a single histogram (which describes the distribution of pixel intensity) and derives some basic features (such as energy, entropy, kurtosis, and skewness) from it. Shape-based features describe the geometric structure of ROI, which is useful in a sense because the shape of placental tissue is highly correlated with placental invasion [32, 37]. Texture based features are the most informative features, especially for the issue of tissue heterogeneity, because texture-based features can capture the spatial relationship between adjacent pixels [8, 9, 34]. In this paper, we use the Gray-level co-occurrence matrix (GLCM), Gray-level run length matrix (GLRLM), and Gray-level size zone matrix (GLSZM), etc. to calculate various texture features. We extracted 100 image features, including 18 intensity-based features, 9 shape-based features, and 73 texture-based features.

### Extraction of deep features

**Fig. 6:**
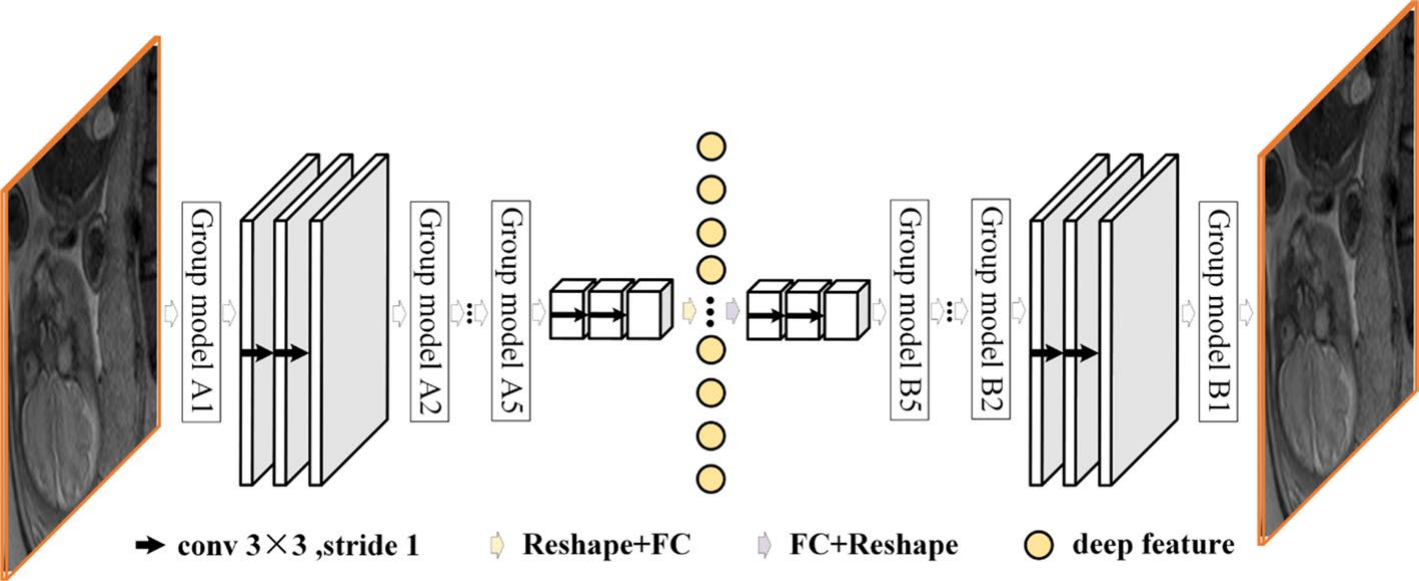
The structure of DDCNN

Radiomic features can describe the gray distribution, shape, texture, and other characteristics of placental tissue in MR images, but it is difficult to accurately describe the overall structural relationship between lesions and surrounding tissues, which is of great significance for the diagnosis of placental invasion and evaluation of the degree of placenta implantation. In recent years, image features extracted by deep convolution neural network (DCNN) have been proved to be effective in improving the accuracy of image classification, segmentation, or retrieval [38]. We transformed the prenatal prediction and typing of placental invasion into a classification problem. Therefore, according to the characteristics of placental invasion in MR images, a deep dynamic convolution neural network (DDCNN) is designed to extract the deep features. The structure of DDCNN is shown in Fig. 6. As can be seen from Fig. 6, the backbone of DDCNN is a multi-layer automatic coding network, which is composed of the encoder and decoder with a symmetrical structure [39]. During network training, we intercept the original MR image with the smallest bounding rectangle of the extracted ROI and use it as the input of DDCNN. In the encoding stage, the input MR image undergoes a 5-stage Group Model A convolution and pooling operation, and the input MR image is mapped into a feature vector that can represent the semantic information of the placenta. In the decoding stage, the feature vector output by the encoder undergoes a 5-stage Group Model B upsampling and deconvolution operation and restores the original input image as much as possible as the training target. After the DDCNN training is completed, we remove the decoder, fix the encoder parameters, and input the smallest external rectangle region containing the ROI of the MR image, then we can project it into a low-order feature space through the encoder, and realize the extraction of depth features. With this structure, DDCNN can extract the depth features of MR images through unsupervised training, thus solving the problems of traditional CNN in extracting the depth features of MR images, such as the difficulty of extracting supervised samples and the network easily falling into overfitting.

**Fig. 7:**
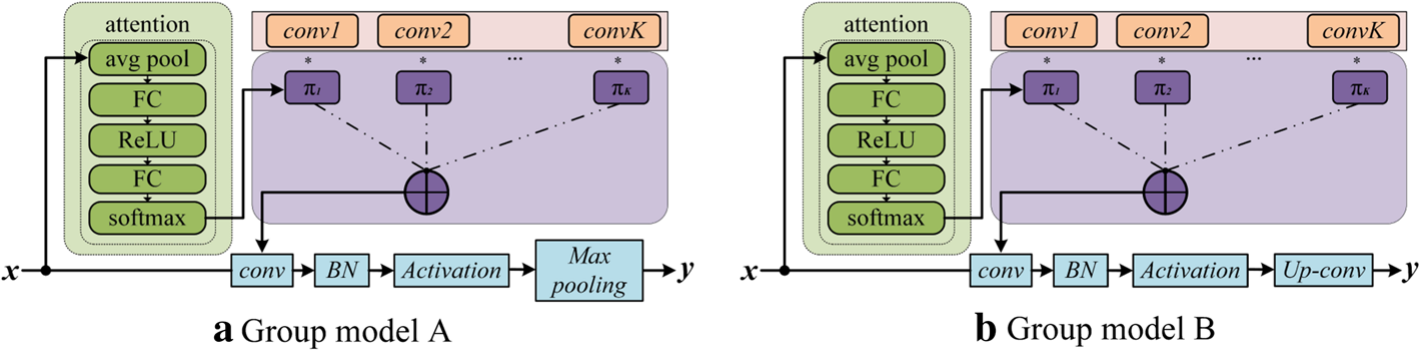
The structure of the Group module

The Group Model A and Group Model B of the DDCNN are shown in Fig. 7. Each level of the Group Model is mainly composed of a dynamic convolutional layer [40], RELU activation layer [41], BN layer [42], and the max-pooling layer or up-sampling layer.

It can be seen from Fig. 7 that in the Group Model, the traditional convolution operation is replaced by dynamic convolution. The method to achieve dynamic convolution is to replace the traditional fixed convolution kernel with a dynamic convolution kernel that is adaptively adjusted with the input image. Specifically, it uses the mechanism of multiple convolution kernels and introduces a lightweight squeeze and excitation module [30] to build an attention model. Through model training, the respective weights of multiple convolution kernels are obtained, and the dynamic convolution kernel obtained by weighting and superimposing each convolution kernel participates in the convolution operation. Suppose the multiple convolution kernels introduced in a certain layer of convolution are ${\text{conv}}_1,{\text{conv}}_2,\ldots ,covn_N$, and their respective weights are $w_1,w_2,\ldots ,w_N$. The squeeze and excitation introduced in the model is shown in the attention module in Fig. 7. The input images are processed by average pooling, full connection, relu, and finally mapped to the output $w_1,w_2,\ldots ,w_N$ by softmax [40]. Where:

8 $$\begin{aligned} w_1+w_2+\ldots +w_N=1 \end{aligned}$$

where *N* denotes the number of convolution kernels.

After the weights of each convolution kernel are obtained, the dynamic convolution kernel involved in feature extraction can be constructed through weighted superposition [40]:

9 $$\begin{aligned} {\text{conv}}=\sum _{i=1}^{N}{w_i*{\text{conv}}_i} \end{aligned}$$

where ${\text{conv}}_i$ denotes the ith convolutional kernel, $w_i$ represents the weight of the corresponding ith convolution generated by the attention module, *N* indicates the number of convolutional kernels, and *conv* is the synthesized convolutional kernel, which means the final convolutional kernel involved in the operation in Group Model A.

With this structure, the convolution kernel will be adjusted adaptively with the input image during convolution operation, which can better adapt to the structural heterogeneity of placenta tissue of different patients and different types of placental invasion, so that the extracted deep features can effectively describe the pathological information contained in placental tissue.

### Prenatal prediction and typing of placental invasion

**Fig. 8:**
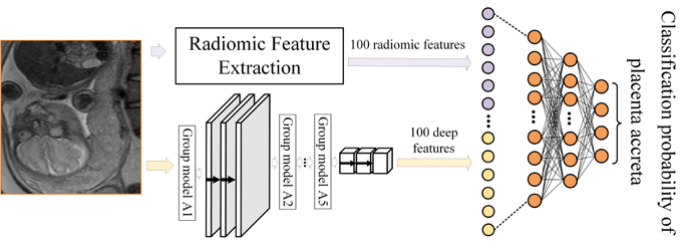
Classification framework that combines deep with radiomic features

Based on the above-mentioned features, we train a classifier using the multi-layer perceptron model to divide the patients into four types: no placental invasion, placenta accreta, placenta increta, and placenta percreta according to the T2WI images, so as to realize the prenatal prediction and typing of placental invasion. The constructed classifier is shown in Fig. 8. As shown in Fig. 8, the input of the classifier is the radiomic feature extracted from the ROI and the deep features extracted by the DDCNN encoder. To maintain the balance between the two types of features, their dimensions are all set to 100. When training the classifier, the results confirmed by clinical or surgical pathology are used as supervision information. The classifier consists of four layers, in which the number of neurons in each layer is 200, 100, 20, and 4 respectively. The activation function of the middle layers is Relu, and the output of the last layer of the classifier is a 4-dimensional feature vector, which is activated by softmax which is often used in multi-classification problems.

The softmax [40] first enhances the difference between input values by nonlinear exponential operation with base *exp*, and then the output of multiple neurons is mapped to the values in the (0,1) interval and normalized to a probability distribution, so as to perform multi-classification, as shown in formula (10).

10 $$\begin{aligned} {S_i=\frac{\exp ^{y_{j}}}{\sum _{j=0}^{3}{exp^{y_{j}} }}},{0<=i<=3} \end{aligned}$$

where $y_j$ is the output of the classifier, $S_i$ is the probability value of patients corresponding to four types of no placental invasion, placenta accreta, placenta increta, and placenta percreta, and the type with the highest probability is taken as the final prediction result.

## Data Availability

As these data involve personal privacy of patients, it has not been made public yet.

## Abbreviations

PI: Placental invasion
MR: Magnetic resonance
ROI: Region of interest
DDCNN: Deep dynamic convolution neural network
AUC: The area under the ROC curve
PA: Placenta accrete
PC: Placenta increta
PP: Placenta percreta
DIC: Diffuse intravascular coagulation
US: The color Doppler ultrasound
MRI: Magnetic resonance imaging
CNN: The deep convolution neural network
PACS: Picture archiving and communication system
DICOM: Digital imaging and communications in medicine
T2WI: T2 weighted image
GLCM: Gray-level co-occurrence matrix
GLRLM: Gray-level run length matrix
GLSZM: Gray-level size zone matrix
ACC: Accuracy
PRE: Precision;
REC: Recall
F1: F1 score
AACC: Average accuracy
ASEN: Average sensitivity
ASPE: Average specificity
ML: Machine learning
DL: Deep learning
RF: Random forest
DT: Decision tree
LR: Logistic regression
ROC: Receiver operating characteristic curve
